# Ethyl 4-(4-cyano­phen­yl)-6-methyl-2-thioxo-1,2,3,4-tetra­hydro­pyrimidine-5-carboxyl­ate

**DOI:** 10.1107/S1600536809024520

**Published:** 2009-07-01

**Authors:** De-Hong Wu, You-Hong Zhang, Zhu-Feng Li

**Affiliations:** a Ordered Matter Science Research Center, College of Chemistry and Chemical Engineering, School of Materials Science and Engineering, Southeast University, Nanjing 210096, People’s Republic of China

## Abstract

The asymmetric unit of the title compound, C_15_H_15_N_3_O_2_S, contains two independent mol­ecules corresponding to the *R* and *S* enanti­omers. The dihydro­pyrimidinone rings adopt a flattened boat conformation. One of the ethyl groups is disordered over two orientations with occupancy factors of 0.700 (7) and 0.300 (7). In the crystal structure, mol­ecules are linked by inter­molecular N—H⋯O hydrogen-bonding inter­actions into one-dimensional chains along the *c*-axis direction. The chains are further connected by N—H⋯S hydrogen bonds, forming a three-dimensional network.

## Related literature

For the synthesis and the pharmaceutical applications of pyrimidinones, see: Atwal (1990 ▶); Steele *et al.* (1998 ▶); Manjula *et al.* (2004 ▶); Matsuda & Hirao (1965 ▶).
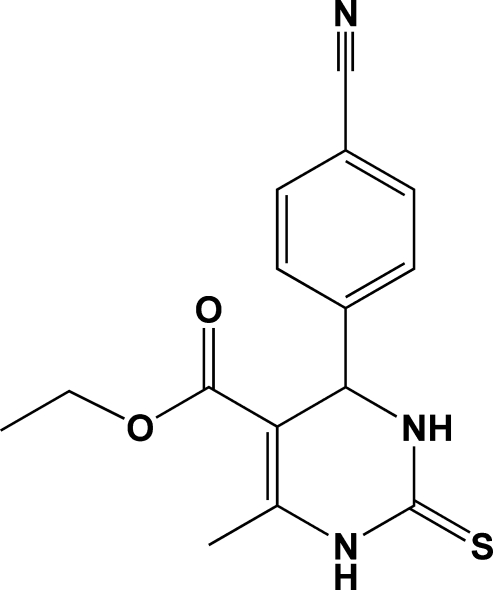


## Experimental

### 

#### Crystal data

C_15_H_15_N_3_O_2_S
*M* *_r_* = 301.37
Triclinic, 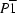

*a* = 9.2938 (19) Å
*b* = 13.277 (3) Å
*c* = 14.512 (3) Å
α = 101.247 (17)°
β = 108.442 (13)°
γ = 107.89 (3)°
*V* = 1529.6 (7) Å^3^
*Z* = 4
Mo *K*α radiation
μ = 0.22 mm^−1^
*T* = 291 K
0.50 × 0.48 × 0.47 mm

#### Data collection

Rigaku SCXmini diffractometer
Absorption correction: multi-scan (*CrystalClear*; Rigaku, 2005 ▶) *T*_min_ = 0.898, *T*_max_ = 0.904
13899 measured reflections
5958 independent reflections
4590 reflections with *I* > 2σ(*I*)
*R*_int_ = 0.029

#### Refinement

*R*[*F* ^2^ > 2σ(*F* ^2^)] = 0.050
*wR*(*F* ^2^) = 0.151
*S* = 1.06
5958 reflections
386 parameters
2 restraints
H-atom parameters constrained
Δρ_max_ = 0.32 e Å^−3^
Δρ_min_ = −0.30 e Å^−3^

### 

Data collection: *CrystalClear* (Rigaku, 2005 ▶); cell refinement: *CrystalClear*; data reduction: *CrystalClear*; program(s) used to solve structure: *SHELXS97* (Sheldrick, 2008 ▶); program(s) used to refine structure: *SHELXL97* (Sheldrick, 2008 ▶); molecular graphics: *SHELXTL* (Sheldrick, 2008 ▶); software used to prepare material for publication: *SHELXTL*.

## Supplementary Material

Crystal structure: contains datablocks I, global. DOI: 10.1107/S1600536809024520/rz2341sup1.cif

Structure factors: contains datablocks I. DOI: 10.1107/S1600536809024520/rz2341Isup2.hkl

Additional supplementary materials:  crystallographic information; 3D view; checkCIF report

## Figures and Tables

**Table 1:** Hydrogen-bond geometry (Å, °)

*D*—H⋯*A*	*D*—H	H⋯*A*	*D*⋯*A*	*D*—H⋯*A*
N1—H1*A*⋯S2^i^	0.86	2.60	3.4612 (19)	174
N2—H2*A*⋯O3^ii^	0.86	2.14	2.843 (2)	138
N5—H5*A*⋯O1^iii^	0.86	2.01	2.852 (2)	165

Symmetry codes: (i) 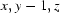; (ii) 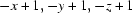; (iii) 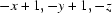.

**Fig. 1.**
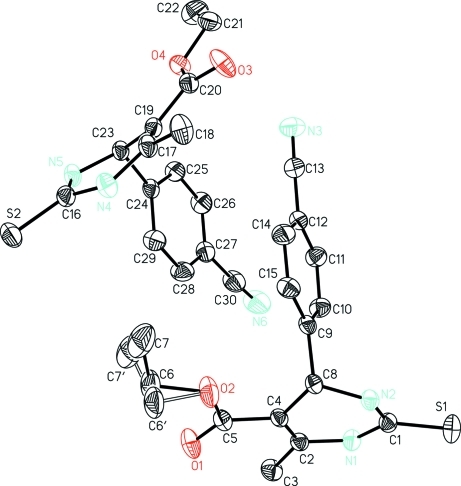
The content of asymmetric unit of the title compound showing the atom numbering scheme and displacement ellipsoids drawn at the 30% probability level. H atoms are omitted for clarity.

**Fig. 2.**
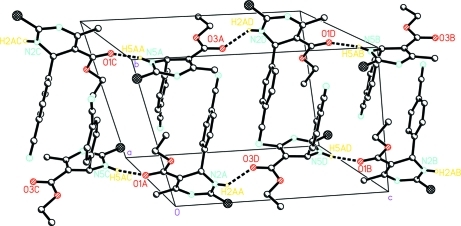
The crystal packing of the title compound, showing the chains along [001] formed by N—H···O hydrogen bonds (dashed lines). The minor component of disorder and H atoms not involved in hydrogen bonds are omitted for clarity.

## supplementary crystallographic information

### Comment

The common synthetic routes to the synthesis of dihydropyrimidinone derivatives generally involve multi-step transformations that are essentially based on the Biginelli condensation methodology (Steele *et al.*, 1998). 3,4-Dihydropyrimidinones are compounds which have drawn wide-spread attention due to their pharmaceutical applications. A variety of dihydropyrimidinone derivatives have been screened for antihypertension (Atwal, 1990), antibacterial (Matsuda & Hirao, 1965) and calcium channel blocking (Manjula *et al.*, 2004) activities.We report herein the crystal structure of the title compound, ethyl 4-(4-cyanophenyl)-6-methyl-2-thioxo-1,2,3,4-tetrahydro-pyrimidine-5-carboxylate (Fig. 1).

The asymmetric unit of the title compound contains two independent molecules corresponding to the *R*- and *S*-enantiomers. One ethyl group (C6–C7) is disordered over two orientations with refined occupancy factors of 0.700 (7) and 0.300 (7). The dihydropyrimidinone rings adopt a flattened boat conformation. In the crystal structure, the molecules are linked by intermolecular N—H···O hydrogen bonding interactions (Table 1) into one-dimensional chains along the *c* direction (Fig. 2). The chains are further connected by N—H···S hydrogen bonds forming a three-dimensional network.

### Experimental

The title compound was synthesized by refluxing 4-cyanobenzaldehyde (2 mmol), ethyl acetoacetate (2 mmol), thiourea (3 mmol) and NH_4_Cl (1 mmol) in acetic acid (10 ml) at 100 °C for 8 h. The reaction mixture was then allowed to stand at room temperature and the product formed was filtered, washed with ethanol followed by water and dried. Further purification was done by recrystallization from ethanol. Single crystals suitable for X-ray analysis were obtained by slow evaporation of an ethanol solution for 4 weeks.

### Refinement

H atoms were placed in calculated positions (N—H = 0.86 Å; C—H = 0.93–0.98 Å for C*sp^2^* and C*sp^3^* atoms, respectively), assigned fixed *U*_iso_ values [*U*_iso_ = 1.2*U*eq(C*sp^2^*/N) and 1.5*U*eq(C*sp^3^*)] and allowed to ride. The ethyl group labeled by C(6) and C(7) is disordered over two positions with occupancies of 0.700 (7) and 0.300 (7), and all disordered atoms were subjected to a rigid bond restraint.

### Crystal data

**Table:**

C_15_H_15_N_3_O_2_S	*Z* = 4
*M**_r_* = 301.37	*F*(000) = 632
Triclinic, *P*1	*D*_x_ = 1.309 Mg m^−^^3^
Hall symbol: -P 1	Mo *K*α radiation, λ = 0.71073 Å
*a* = 9.2938 (19) Å	Cell parameters from 4203 reflections
*b* = 13.277 (3) Å	θ = 2.4–27.5°
*c* = 14.512 (3) Å	µ = 0.22 mm^−^^1^
α = 101.247 (17)°	*T* = 291 K
β = 108.442 (13)°	Block, yellow
γ = 107.89 (3)°	0.50 × 0.48 × 0.47 mm
*V* = 1529.6 (7) Å^3^	

### Data collection

**Table:**

Rigaku SCXmini diffractometer	5958 independent reflections
Radiation source: fine-focus sealed tube	4590 reflections with *I* > 2σ(*I*)
graphite	*R*_int_ = 0.029
Detector resolution: 13.6612 pixels mm^-1^	θ_max_ = 26.0°, θ_min_ = 2.4°
ω scans	*h* = −11→11
Absorption correction: multi-scan (*CrystalClear*; Rigaku, 2005)	*k* = −16→16
*T*_min_ = 0.898, *T*_max_ = 0.904	*l* = −17→17
13899 measured reflections	

### Refinement

**Table:**

Refinement on *F*^2^	Primary atom site location: structure-invariant direct methods
Least-squares matrix: full	Secondary atom site location: difference Fourier map
*R*[*F*^2^ > 2σ(*F*^2^)] = 0.050	Hydrogen site location: inferred from neighbouring sites
*wR*(*F*^2^) = 0.151	H-atom parameters constrained
*S* = 1.06	*w* = 1/[σ^2^(*F*_o_^2^) + (0.0841*P*)^2^ + 0.2227*P*] where *P* = (*F*_o_^2^ + 2*F*_c_^2^)/3
5958 reflections	(Δ/σ)_max_ < 0.001
386 parameters	Δρ_max_ = 0.32 e Å^−^^3^
2 restraints	Δρ_min_ = −0.30 e Å^−^^3^

### Special details

**Table:**

Experimental. 1*H*-NMR (d4-Methanol) δ (p.p.m.): 7.73 (d, 2H, J = 8 Hz), 7.50 (d, 2H, J = 8 Hz), 5.40 (s, 1H), 4.11 (q, 2H, J = 7 Hz), 2.37 (s, 3H), 1.19(t, 3H, J = 7 Hz). 13 C-NMR (d4-Methanol) δ(p.p.m.): 13.08 (–CH2—CH3), 16.33 (–CH3), 54.66 (C*), 60.03 (–CH2—CH3), 100.73 (C—C=O), 111.32 (C—CN), 118.08 (–CN), 127.46, 132.29 (CH in phenyl), 145.42 (C in phenyl), 148.56 (CH3—C—NH–), 165.53 (C=O), 175.35 (C=S).
Geometry. All e.s.d.'s (except the e.s.d. in the dihedral angle between two l.s. planes) are estimated using the full covariance matrix. The cell e.s.d.'s are taken into account individually in the estimation of e.s.d.'s in distances, angles and torsion angles; correlations between e.s.d.'s in cell parameters are only used when they are defined by crystal symmetry. An approximate (isotropic) treatment of cell e.s.d.'s is used for estimating e.s.d.'s involving l.s. planes.
Refinement. Refinement of *F*^2^ against ALL reflections. The weighted *R*-factor *wR* and goodness of fit *S* are based on *F*^2^, conventional *R*-factors *R* are based on *F*, with *F* set to zero for negative *F*^2^. The threshold expression of *F*^2^ > σ(*F*^2^) is used only for calculating *R*-factors(gt) *etc*. and is not relevant to the choice of reflections for refinement. *R*-factors based on *F*^2^ are statistically about twice as large as those based on *F*, and *R*- factors based on ALL data will be even larger.

### Fractional atomic coordinates and isotropic or equivalent isotropic displacement parameters (Å^2^)

**Table:**

	*x*	*y*	*z*	*U*_iso_*/*U*_eq_	Occ. (<1)
C1	0.2143 (2)	−0.01219 (17)	0.21806 (15)	0.0419 (4)	
C2	0.2370 (2)	0.03499 (16)	0.06791 (14)	0.0398 (4)	
C3	0.2591 (3)	−0.0104 (2)	−0.02697 (17)	0.0561 (6)	
H3A	0.2465	0.0359	−0.0696	0.084*	
H3B	0.1776	−0.0852	−0.0639	0.084*	
H3C	0.3675	−0.0110	−0.0084	0.084*	
C4	0.2051 (2)	0.12633 (16)	0.09358 (13)	0.0374 (4)	
C5	0.1790 (3)	0.19201 (18)	0.02444 (15)	0.0454 (5)	
C6	0.1450 (8)	0.3589 (6)	0.0092 (7)	0.082 (2)	0.700 (7)
H6A	0.0435	0.3271	−0.0523	0.098*	0.700 (7)
H6B	0.2375	0.3768	−0.0108	0.098*	0.700 (7)
C7	0.1533 (8)	0.4606 (4)	0.0772 (5)	0.1006 (17)	0.700 (7)
H7A	0.1462	0.5134	0.0410	0.151*	0.700 (7)
H7B	0.2560	0.4930	0.1366	0.151*	0.700 (7)
H7C	0.0632	0.4415	0.0983	0.151*	0.700 (7)
C8	0.2021 (2)	0.16692 (16)	0.19771 (13)	0.0368 (4)	
H8A	0.1093	0.1899	0.1884	0.044*	
C9	0.3609 (2)	0.26699 (16)	0.27233 (14)	0.0387 (4)	
C10	0.5112 (3)	0.25592 (17)	0.30151 (16)	0.0474 (5)	
H10A	0.5152	0.1881	0.2735	0.057*	
C11	0.6553 (3)	0.34466 (18)	0.37184 (17)	0.0510 (5)	
H11A	0.7548	0.3357	0.3922	0.061*	
C12	0.6507 (3)	0.44634 (17)	0.41161 (15)	0.0461 (5)	
C13	0.8014 (3)	0.53885 (19)	0.48441 (17)	0.0554 (6)	
C14	0.5017 (3)	0.45957 (19)	0.38096 (17)	0.0554 (6)	
H14A	0.4989	0.5286	0.4064	0.067*	
C15	0.3576 (3)	0.36968 (18)	0.31243 (16)	0.0495 (5)	
H15A	0.2578	0.3782	0.2931	0.059*	
C16	0.5233 (3)	0.77227 (17)	0.18205 (15)	0.0451 (5)	
C17	0.5666 (3)	0.77320 (17)	0.35679 (15)	0.0436 (5)	
C18	0.4795 (3)	0.7769 (2)	0.42837 (19)	0.0603 (6)	
H18A	0.5549	0.7901	0.4967	0.091*	
H18B	0.3873	0.7069	0.4060	0.091*	
H18C	0.4408	0.8362	0.4280	0.091*	
C19	0.7254 (2)	0.78795 (16)	0.38041 (14)	0.0384 (4)	
C20	0.8419 (3)	0.82399 (17)	0.48790 (15)	0.0439 (5)	
C21	1.1249 (3)	0.9003 (2)	0.60221 (18)	0.0630 (6)	
H21A	1.1071	0.8446	0.6364	0.076*	
H21B	1.1211	0.9669	0.6412	0.076*	
C22	1.2875 (3)	0.9271 (3)	0.5945 (3)	0.0823 (9)	
H22A	1.3741	0.9563	0.6621	0.123*	
H22B	1.3032	0.9818	0.5601	0.123*	
H22C	1.2900	0.8604	0.5562	0.123*	
C23	0.7893 (2)	0.77481 (16)	0.29625 (14)	0.0382 (4)	
H23A	0.9001	0.8337	0.3216	0.046*	
C24	0.8018 (2)	0.66181 (16)	0.26707 (14)	0.0381 (4)	
C25	0.9496 (3)	0.65221 (18)	0.31451 (17)	0.0488 (5)	
H25A	1.0406	0.7151	0.3626	0.059*	
C26	0.9640 (3)	0.5505 (2)	0.29138 (18)	0.0556 (6)	
H26A	1.0641	0.5455	0.3238	0.067*	
C27	0.8294 (3)	0.45617 (18)	0.21999 (16)	0.0504 (5)	
C28	0.6818 (3)	0.4646 (2)	0.17110 (18)	0.0591 (6)	
H28A	0.5913	0.4017	0.1226	0.071*	
C29	0.6684 (3)	0.56674 (19)	0.19433 (17)	0.0538 (5)	
H29A	0.5687	0.5718	0.1608	0.065*	
C30	0.8447 (4)	0.3498 (2)	0.20089 (19)	0.0659 (7)	
C6'	0.070 (2)	0.3356 (16)	−0.0032 (18)	0.082 (2)	0.300 (7)
H6'A	0.0324	0.2960	−0.0756	0.098*	0.300 (7)
H6'B	−0.0202	0.3477	0.0105	0.098*	0.300 (7)
C7'	0.2159 (17)	0.4385 (11)	0.0334 (12)	0.1006 (17)	0.300 (7)
H7'A	0.1932	0.4860	−0.0063	0.151*	0.300 (7)
H7'B	0.3057	0.4212	0.0264	0.151*	0.300 (7)
H7'C	0.2453	0.4762	0.1043	0.151*	0.300 (7)
N1	0.2529 (2)	−0.02691 (14)	0.13483 (13)	0.0448 (4)	
H1A	0.2896	−0.0779	0.1228	0.054*	
N2	0.1754 (2)	0.07505 (14)	0.24092 (12)	0.0416 (4)	
H2A	0.1310	0.0779	0.2846	0.050*	
N3	0.9225 (3)	0.60999 (18)	0.54293 (17)	0.0738 (6)	
N4	0.4650 (2)	0.75428 (16)	0.25562 (13)	0.0516 (4)	
H4A	0.3600	0.7300	0.2385	0.062*	
N5	0.6813 (2)	0.79053 (14)	0.20621 (12)	0.0441 (4)	
H5A	0.7242	0.8135	0.1658	0.053*	
N6	0.8613 (4)	0.2675 (2)	0.1898 (2)	0.0943 (9)	
O1	0.1803 (2)	0.17349 (17)	−0.06000 (12)	0.0695 (5)	
O2	0.1501 (3)	0.27880 (16)	0.06545 (13)	0.0776 (6)	
O3	0.8045 (2)	0.82617 (18)	0.56068 (12)	0.0720 (5)	
O4	0.99902 (18)	0.85749 (13)	0.49825 (11)	0.0513 (4)	
S1	0.21451 (8)	−0.10085 (6)	0.28636 (5)	0.0632 (2)	
S2	0.39778 (8)	0.77075 (5)	0.06890 (4)	0.0592 (2)	

### Atomic displacement parameters (Å^2^)

**Table:**

	*U*^11^	*U*^22^	*U*^33^	*U*^12^	*U*^13^	*U*^23^
C1	0.0376 (10)	0.0473 (11)	0.0412 (10)	0.0142 (8)	0.0170 (9)	0.0176 (9)
C2	0.0396 (10)	0.0435 (10)	0.0339 (9)	0.0131 (8)	0.0160 (8)	0.0111 (8)
C3	0.0722 (15)	0.0587 (13)	0.0437 (11)	0.0300 (12)	0.0304 (11)	0.0110 (10)
C4	0.0373 (9)	0.0438 (10)	0.0305 (9)	0.0142 (8)	0.0148 (8)	0.0124 (8)
C5	0.0460 (11)	0.0568 (12)	0.0369 (10)	0.0220 (9)	0.0173 (9)	0.0190 (9)
C6	0.119 (6)	0.075 (4)	0.075 (3)	0.057 (5)	0.035 (5)	0.048 (3)
C7	0.114 (4)	0.074 (3)	0.136 (5)	0.051 (3)	0.050 (3)	0.055 (3)
C8	0.0386 (10)	0.0442 (10)	0.0329 (9)	0.0192 (8)	0.0169 (8)	0.0148 (8)
C9	0.0448 (10)	0.0440 (10)	0.0309 (9)	0.0189 (9)	0.0181 (8)	0.0131 (8)
C10	0.0467 (11)	0.0404 (10)	0.0530 (12)	0.0197 (9)	0.0173 (10)	0.0116 (9)
C11	0.0435 (11)	0.0491 (12)	0.0551 (13)	0.0174 (10)	0.0147 (10)	0.0161 (10)
C12	0.0517 (12)	0.0451 (11)	0.0353 (10)	0.0122 (9)	0.0166 (9)	0.0130 (8)
C13	0.0643 (14)	0.0469 (12)	0.0485 (12)	0.0175 (11)	0.0198 (12)	0.0145 (10)
C14	0.0690 (15)	0.0440 (12)	0.0513 (12)	0.0263 (11)	0.0225 (11)	0.0079 (9)
C15	0.0513 (12)	0.0517 (12)	0.0448 (11)	0.0264 (10)	0.0163 (10)	0.0094 (9)
C16	0.0611 (13)	0.0422 (11)	0.0392 (10)	0.0269 (10)	0.0216 (10)	0.0157 (8)
C17	0.0533 (12)	0.0497 (11)	0.0409 (10)	0.0265 (9)	0.0260 (9)	0.0204 (9)
C18	0.0621 (14)	0.0887 (18)	0.0607 (14)	0.0418 (13)	0.0427 (12)	0.0379 (13)
C19	0.0518 (11)	0.0390 (10)	0.0373 (10)	0.0246 (9)	0.0247 (9)	0.0166 (8)
C20	0.0581 (12)	0.0448 (11)	0.0386 (10)	0.0285 (10)	0.0237 (10)	0.0138 (8)
C21	0.0676 (15)	0.0553 (14)	0.0467 (12)	0.0228 (12)	0.0068 (11)	0.0058 (10)
C22	0.0594 (16)	0.0692 (17)	0.095 (2)	0.0237 (13)	0.0063 (15)	0.0221 (15)
C23	0.0468 (10)	0.0412 (10)	0.0361 (9)	0.0211 (8)	0.0222 (8)	0.0168 (8)
C24	0.0490 (11)	0.0434 (10)	0.0343 (9)	0.0236 (9)	0.0247 (9)	0.0161 (8)
C25	0.0502 (12)	0.0504 (12)	0.0488 (12)	0.0250 (10)	0.0195 (10)	0.0152 (9)
C26	0.0582 (13)	0.0606 (14)	0.0585 (13)	0.0369 (12)	0.0233 (11)	0.0197 (11)
C27	0.0710 (14)	0.0501 (12)	0.0446 (11)	0.0367 (11)	0.0286 (11)	0.0159 (9)
C28	0.0675 (15)	0.0488 (13)	0.0532 (13)	0.0262 (11)	0.0187 (12)	0.0043 (10)
C29	0.0532 (12)	0.0535 (12)	0.0537 (13)	0.0290 (10)	0.0160 (11)	0.0114 (10)
C30	0.0886 (18)	0.0633 (15)	0.0518 (13)	0.0462 (14)	0.0248 (13)	0.0111 (11)
C6'	0.119 (6)	0.075 (4)	0.075 (3)	0.057 (5)	0.035 (5)	0.048 (3)
C7'	0.114 (4)	0.074 (3)	0.136 (5)	0.051 (3)	0.050 (3)	0.055 (3)
N1	0.0559 (10)	0.0441 (9)	0.0463 (9)	0.0253 (8)	0.0274 (8)	0.0189 (7)
N2	0.0458 (9)	0.0482 (9)	0.0372 (8)	0.0181 (8)	0.0237 (7)	0.0162 (7)
N3	0.0695 (14)	0.0556 (12)	0.0631 (13)	0.0094 (11)	0.0093 (12)	0.0049 (10)
N4	0.0486 (10)	0.0679 (12)	0.0459 (10)	0.0265 (9)	0.0219 (8)	0.0240 (9)
N5	0.0591 (10)	0.0524 (10)	0.0398 (9)	0.0317 (8)	0.0288 (8)	0.0238 (8)
N6	0.134 (2)	0.0732 (16)	0.0725 (15)	0.0687 (17)	0.0222 (16)	0.0050 (12)
O1	0.0948 (13)	0.1007 (14)	0.0499 (9)	0.0593 (11)	0.0437 (9)	0.0432 (9)
O2	0.1407 (18)	0.0738 (12)	0.0546 (10)	0.0714 (13)	0.0467 (11)	0.0369 (9)
O3	0.0800 (12)	0.1160 (15)	0.0370 (8)	0.0530 (11)	0.0314 (8)	0.0239 (9)
O4	0.0519 (9)	0.0556 (9)	0.0404 (8)	0.0189 (7)	0.0153 (7)	0.0122 (6)
S1	0.0743 (4)	0.0735 (4)	0.0747 (4)	0.0400 (3)	0.0448 (4)	0.0500 (4)
S2	0.0743 (4)	0.0698 (4)	0.0399 (3)	0.0421 (3)	0.0167 (3)	0.0196 (3)

### Geometric parameters (Å, °)

**Table:**

C1—N2	1.329 (3)	C18—H18A	0.9600
C1—N1	1.361 (2)	C18—H18B	0.9600
C1—S1	1.680 (2)	C18—H18C	0.9600
C2—C4	1.346 (3)	C19—C20	1.469 (3)
C2—N1	1.391 (3)	C19—C23	1.521 (2)
C2—C3	1.494 (3)	C20—O3	1.210 (2)
C3—H3A	0.9600	C20—O4	1.337 (3)
C3—H3B	0.9600	C21—O4	1.452 (3)
C3—H3C	0.9600	C21—C22	1.489 (4)
C4—C5	1.464 (3)	C21—H21A	0.9700
C4—C8	1.514 (2)	C21—H21B	0.9700
C5—O1	1.206 (2)	C22—H22A	0.9600
C5—O2	1.333 (3)	C22—H22B	0.9600
C6—O2	1.464 (8)	C22—H22C	0.9600
C6—C7	1.473 (9)	C23—N5	1.471 (2)
C6—H6A	0.9700	C23—C24	1.528 (3)
C6—H6B	0.9700	C23—H23A	0.9800
C7—H7A	0.9600	C24—C25	1.386 (3)
C7—H7B	0.9600	C24—C29	1.391 (3)
C7—H7C	0.9600	C25—C26	1.385 (3)
C8—N2	1.467 (2)	C25—H25A	0.9300
C8—C9	1.529 (3)	C26—C27	1.385 (3)
C8—H8A	0.9800	C26—H26A	0.9300
C9—C15	1.388 (3)	C27—C28	1.381 (3)
C9—C10	1.389 (3)	C27—C30	1.445 (3)
C10—C11	1.386 (3)	C28—C29	1.385 (3)
C10—H10A	0.9300	C28—H28A	0.9300
C11—C12	1.380 (3)	C29—H29A	0.9300
C11—H11A	0.9300	C30—N6	1.137 (3)
C12—C14	1.391 (3)	C6'—C7'	1.453 (16)
C12—C13	1.445 (3)	C6'—O2	1.51 (2)
C13—N3	1.144 (3)	C6'—H6'A	0.9700
C14—C15	1.386 (3)	C6'—H6'B	0.9700
C14—H14A	0.9300	C7'—H7'A	0.9600
C15—H15A	0.9300	C7'—H7'B	0.9600
C16—N5	1.327 (3)	C7'—H7'C	0.9600
C16—N4	1.370 (3)	N1—H1A	0.8600
C16—S2	1.679 (2)	N2—H2A	0.8600
C17—C19	1.345 (3)	N4—H4A	0.8600
C17—N4	1.394 (3)	N5—H5A	0.8600
C17—C18	1.508 (3)		
			
N2—C1—N1	116.14 (17)	C20—C19—C23	118.95 (17)
N2—C1—S1	122.57 (15)	O3—C20—O4	122.34 (19)
N1—C1—S1	121.29 (16)	O3—C20—C19	125.4 (2)
C4—C2—N1	118.89 (17)	O4—C20—C19	112.29 (16)
C4—C2—C3	127.33 (19)	O4—C21—C22	107.1 (2)
N1—C2—C3	113.77 (18)	O4—C21—H21A	110.3
C2—C3—H3A	109.5	C22—C21—H21A	110.3
C2—C3—H3B	109.5	O4—C21—H21B	110.3
H3A—C3—H3B	109.5	C22—C21—H21B	110.3
C2—C3—H3C	109.5	H21A—C21—H21B	108.5
H3A—C3—H3C	109.5	C21—C22—H22A	109.5
H3B—C3—H3C	109.5	C21—C22—H22B	109.5
C2—C4—C5	121.66 (17)	H22A—C22—H22B	109.5
C2—C4—C8	120.44 (17)	C21—C22—H22C	109.5
C5—C4—C8	117.85 (17)	H22A—C22—H22C	109.5
O1—C5—O2	121.6 (2)	H22B—C22—H22C	109.5
O1—C5—C4	126.9 (2)	N5—C23—C19	109.13 (15)
O2—C5—C4	111.51 (17)	N5—C23—C24	110.81 (15)
O2—C6—C7	108.0 (6)	C19—C23—C24	112.27 (15)
O2—C6—H6A	110.1	N5—C23—H23A	108.2
C7—C6—H6A	110.1	C19—C23—H23A	108.2
O2—C6—H6B	110.1	C24—C23—H23A	108.2
C7—C6—H6B	110.1	C25—C24—C29	118.28 (19)
H6A—C6—H6B	108.4	C25—C24—C23	119.63 (18)
C6—C7—H7A	109.5	C29—C24—C23	122.09 (18)
C6—C7—H7B	109.5	C26—C25—C24	121.0 (2)
H7A—C7—H7B	109.5	C26—C25—H25A	119.5
C6—C7—H7C	109.5	C24—C25—H25A	119.5
H7A—C7—H7C	109.5	C25—C26—C27	120.0 (2)
H7B—C7—H7C	109.5	C25—C26—H26A	120.0
N2—C8—C4	109.60 (15)	C27—C26—H26A	120.0
N2—C8—C9	109.97 (15)	C28—C27—C26	119.6 (2)
C4—C8—C9	112.24 (15)	C28—C27—C30	121.2 (2)
N2—C8—H8A	108.3	C26—C27—C30	119.1 (2)
C4—C8—H8A	108.3	C27—C28—C29	120.0 (2)
C9—C8—H8A	108.3	C27—C28—H28A	120.0
C15—C9—C10	118.92 (18)	C29—C28—H28A	120.0
C15—C9—C8	120.95 (17)	C28—C29—C24	121.0 (2)
C10—C9—C8	120.14 (17)	C28—C29—H29A	119.5
C11—C10—C9	120.83 (19)	C24—C29—H29A	119.5
C11—C10—H10A	119.6	N6—C30—C27	177.3 (3)
C9—C10—H10A	119.6	C7'—C6'—O2	95.9 (11)
C12—C11—C10	119.8 (2)	C7'—C6'—H6'A	112.6
C12—C11—H11A	120.1	O2—C6'—H6'A	112.6
C10—C11—H11A	120.1	C7'—C6'—H6'B	112.6
C11—C12—C14	119.99 (19)	O2—C6'—H6'B	112.6
C11—C12—C13	119.6 (2)	H6'A—C6'—H6'B	110.1
C14—C12—C13	120.4 (2)	C6'—C7'—H7'A	109.5
N3—C13—C12	178.1 (3)	C6'—C7'—H7'B	109.5
C15—C14—C12	119.8 (2)	H7'A—C7'—H7'B	109.5
C15—C14—H14A	120.1	C6'—C7'—H7'C	109.5
C12—C14—H14A	120.1	H7'A—C7'—H7'C	109.5
C14—C15—C9	120.6 (2)	H7'B—C7'—H7'C	109.5
C14—C15—H15A	119.7	C1—N1—C2	124.43 (17)
C9—C15—H15A	119.7	C1—N1—H1A	117.8
N5—C16—N4	116.15 (18)	C2—N1—H1A	117.8
N5—C16—S2	123.41 (16)	C1—N2—C8	125.33 (15)
N4—C16—S2	120.44 (17)	C1—N2—H2A	117.3
C19—C17—N4	119.20 (17)	C8—N2—H2A	117.3
C19—C17—C18	127.79 (19)	C16—N4—C17	123.55 (18)
N4—C17—C18	113.00 (18)	C16—N4—H4A	118.2
C17—C18—H18A	109.5	C17—N4—H4A	118.2
C17—C18—H18B	109.5	C16—N5—C23	125.58 (16)
H18A—C18—H18B	109.5	C16—N5—H5A	117.2
C17—C18—H18C	109.5	C23—N5—H5A	117.2
H18A—C18—H18C	109.5	C5—O2—C6	116.0 (4)
H18B—C18—H18C	109.5	C5—O2—C6'	119.6 (10)
C17—C19—C20	120.67 (17)	C20—O4—C21	116.71 (17)
C17—C19—C23	120.26 (17)		

### Hydrogen-bond geometry (Å, °)

**Table:**

*D*—H···*A*	*D*—H	H···*A*	*D*···*A*	*D*—H···*A*
N1—H1A···S2^i^	0.86	2.60	3.4612 (19)	174
N2—H2A···O3^ii^	0.86	2.14	2.843 (2)	138
N5—H5A···O1^iii^	0.86	2.01	2.852 (2)	165

Symmetry codes: (i) *x*, *y*−1, *z*; (ii) −*x*+1, −*y*+1, −*z*+1; (iii) −*x*+1, −*y*+1, −*z*.
